# Study on the Characteristics of Intestinal Flora Composition in Gastric Cancer Patients and Healthy People in the Qinghai-Tibet Plateau

**DOI:** 10.1007/s12010-021-03732-4

**Published:** 2021-11-18

**Authors:** Zilong Zhang, Linghong Zhu, Yanqing Ma, Bo Wang, Caihong Ci, Jingni Zhang, Yunsong Zhou, Chunjiang Dou, Qiaoling Gu, Yan An, Yongmei Lan, Jin Zhao

**Affiliations:** 1 Northwest Minzu University, Lanzhou, 730030 Gansu Province China; 2 Department of Oncosurgery, Qinghai Provincial People’s Hospital, Xining, 810007 Qinghai Province China; 3 Department of Science and Education, Qinghai Provincial People’s Hospital, Xining, 810007 Qinghai Province China; 4 Key Laboratory of Environmental Ecology and Population Health in Northwest Minority Areas, Northwest Minzu University, Lanzhou, 730030 Gansu Province China

**Keywords:** Qinghai-Tibet Plateau, Gastric cancer, 16S rDNA gene sequencing, Intestinal flora, Diversity

## Abstract

The aim of this study is to compare and analyze the structure and diversity of intestinal flora between gastric cancer patients and healthy people in the Qinghai-Tibet Plateau and to explore the characteristics of the intestinal flora composition in gastric cancer patients in the plateau area, and to determine the possible correlation between the intestinal flora and gastric cancer. Fresh feces from 22 cases of gastric cancer patients diagnosed in a tertiary hospital in Qinghai Province and 30 cases of healthy people during the same period were collected. The 52 subjects were undergone for 16S rDNA gene sequencing of intestinal bacteria to analyze and compare the diversity and compositional characteristics of intestinal flora. Analysis of the diversity of intestinal flora between the gastric cancer group and the healthy group was based on the Chao1 index of species richness, Shannon diversity index, and Simpson index. It showed that the gastric cancer group had no statistically difference from the healthy group (*P* > 0.05). In the Venn diagram, the number of OTU units shared by the gastric cancer group and the healthy group is 6997, and the number of unique OTU units in the healthy group is 2282, while the number of OTU units in the gastric cancer group is 896 and the difference is statistically significant (*χ*^2^ = 495.829), *P* < 0.000). Analysis of the composition and abundance distribution of intestinal flora showed that at the phylum level, there is no significant deference in abundance between the healthy group of Bacteroides and Firmicutes compared with the gastric cancer group (*P* > 0.05). However, there is a statistically significant difference in abundance between the healthy groups of Proteobacteria compared with the gastric cancer group (*P* < 0.05). At the genus level, the gastric cancer group of *Prevotella*_9 is significantly different from the healthy group (*P* < 0.05). Meanwhile, the gastric cancer group of *Streptococcus* and *Lactobacillus* are significantly different from the healthy group (*P* < 0.001). There are differences in the composition and abundance of intestinal flora between patients with gastric cancer and healthy people in plateau areas, suggesting that *Proteobacteria*, *Prevotella*_9, *Streptococcus*, and *Lactobacillus* have increased in the Qinghai-Tibet Plateau and becoming one of the factors related to the incidence of gastric cancer in the region.

## Introduction

The 2020 World Health Organization (WHO) statistical report shows that gastric cancer has become one of the three most common malignant tumors in the world. There are about 480,000 new cases nationwide each year accounting for more than 2/5 of the world, and about 370,000 deaths [1]. The geographical environment of Qinghai Province belongs to the plateau area, and gastric cancer is the malignant tumor with the highest incidence in Qinghai Province [2]. Relevant studies at home and abroad have shown that the occurrence and development of gastric cancer are related to multiple factors such as environment, diet, smoking, genetics, and *Helicobacter pylori* infection. In recent years, the relationship between intestinal flora and disease has become one of the hot research topics. Many studies have shown that the intestinal flora is closely related to various system diseases. However, there are only few studies on the relationship between gastric cancer and intestinal flora in the plateau areas. Qinghai Province is located in the eastern part of the Qinghai-Tibet Plateau, with an average altitude of above 3000 m. Areas above 3000 m above sea level account for 84.1% of the total area of the province. The average temperature in the territory is between − 5.1 and 9.0℃. Qinghai Province has a unique climatic environment, with sparsely populated areas, multi-ethnic communities, inconvenient transportation, economic crisis, and poor mobility of the resident population. The effects of multiple factors have resulted in the formation of a unique diet and living habits in Qinghai. Their diet structure is relatively simple, with more meat and less vegetable; high-calorie, high-fat, and high-salt foods account for a relatively large proportion of their diet and they love drinking alcohol. In this study, patients with gastric cancer and healthy people in Qinghai-Tibet Plateau were used as the research objects. A case–control study was conducted to analyze the compositional characteristics of the intestinal flora of patients with gastric cancer and healthy people. The existence of gut microbiota occurs in the form of biofilm and remains attached to the surface with the help of serine-rich repeats of proteins. We also explore the diversity of intestinal flora and the structural composition of different bacteria genera in patients with gastric malignant tumors who reside in the plateau area, in order to identify the cause of gastric cancer in plateau areas and hence provide new ideas for the treatment of gastric cancer patients, and ultimately reduce the incidence through early intestinal flora intervention.

## Materials and Methods

### General Information

The subjects include 22 patients who were diagnosed with gastric cancer by pathological biopsy and were hospitalized in a tertiary hospital in Qinghai Province from September 2020 to March 2021. Healthy patients are 30 people who came to the hospital physical examination center for health examination at the same time period excluding those with chronic gastritis and acute and chronic gastric diseases such as gastric ulcer.

### Inclusion and Exclusion Criteria

#### Healthy People

They are the adults who have lived in Qinghai Province for more than 15 years and have normal gastric mucosa under gastroscopy, have no gastritis, peptic ulcer, and tumor lesions, have a past physical fitness, and have no digestive system diseases or related symptoms. They also should have no obesity, chronic diabetes, and other endocrine system diseases as well as no history of cardiovascular, respiratory, and other system diseases. No antibiotics, probiotics, antacids, or acid inhibitors were taken within 1 month before sampling, and the breath test was negative.

#### Patients with Gastric Cancer

These are the adults who have lived in Qinghai Province for more than 15 years and found to be a gastric cancer patient for the first time under gastroscopy and confirmed by histopathology. They should have no tumor rupture and bleeding, and those with pyloric obstruction are excluded. No antibiotics, probiotics, antacids, or acid inhibitors were taken within 1 month before sampling, and the breath test was negative.

#### Common Exclusion Criteria

These are the adults who have not lived in Qinghai Province for more than 15 years and have suffered from other digestive system diseases and tumors except gastric cancer, and also those who have gastrointestinal surgery, radiotherapy, and chemotherapy in the past and have been exposed to probiotics and antibiotics in the past 1 month.

### Stool Sample Collection and Pretreatment

The fresh feces of all subjects were collected in the morning and loaded in a sterile container before quickly putting into the ice box and transferred to the laboratory for sub-packaging processing. The fecal sample was accurately weighed at 200 mg before loading into a 2-ml sterile centrifuge tube per sample and stored in a refrigerator at − 80 °C for testing.

### Genomic DNA Extraction and PCR Amplification

**Fig. 1:**
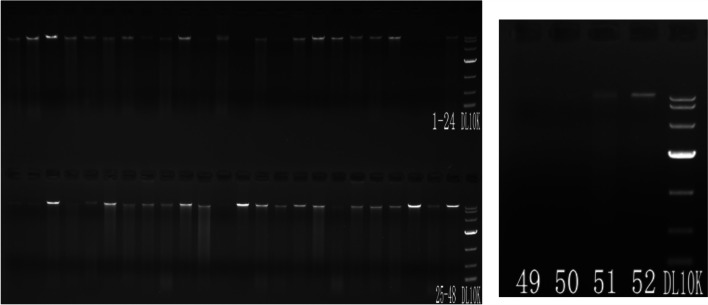
Electrophoresis diagram for detecting the purity and concentration of DNA sample (in 1% agarose gel with 120 V constant pressure electrophoresis for 15 min)

DNA extraction kit (MagPure Soil DNA LQ Kit) was used to extract the genomic DNA of the sample in accordance with the strict instruction. Then, agarose gel electrophoresis was used to detect the purity and concentration of the DNA (see Fig. 1) before taking an appropriate amount of sample in a centrifuge tube, and then, it was diluted with sterile water to 1 ng/μl. The diluted genomic DNA is used as a template for PCR amplification. To ensure amplification efficiency and accuracy, specific primers with barcode and high-fidelity Takara Ex Taq DNA Polymerase (TaKaRa) were used for PCR and were performed in Bio-rad model 580BR10905. The corresponding areas for bacterial diversity identification are as follows: 16S V3-V4 region (forward primer: 343F-5′-TACGGRAGGCAGCAG-3′; reverse primer: 798R-5′-AGGGTATCTAATCCT-3′).

### Mixing and Purification of PCR Products

The PCR product was detected by electrophoresis and purified by magnetic beads. The purified PCR product was used as a PCR template for the second round, and subjected to the second round of PCR amplification, and then detected by electrophoresis again. The PCR product was once again purified by magnetic beads before it was quantified by Qubit. Based on the PCR product concentration, the samples were mixed in the same amount and sequenced on the computer. The original paired-end sequence was generated by sequencing using the Illumina MiSeq platform and the library was constructed. The original sequenced data was uploaded to the database and the sequencing and bioinformatics analysis were completed by Shanghai Ouyi.

### Bioinformatics Analysis Process

The original image data file obtained by high-throughput sequencing is converted into the original sequencing sequence by Base Calling analysis. The results were stored in the FASTQ file format, which contains the information of the sequencing sequence and its corresponding sequencing quality information. Trimmomatic software was used to remove impurity from the original double-ended sequence. At the same time, UCHIME was used to detect and remove the chimera sequence. After the sequencing data was preprocessed to generate high-quality sequences, Vsearch software was used to classify the sequences into multiple OTUs based on the similarity of the sequences. The parameter of an OTU unit is that the sequence similarity is greater than or equal to 97%. The QIIME software package was used to select the representative sequences of each OTU, and compare and annotate all representative sequences in the database. Greengenes or Silva (Version132) database was used for comparison in 16S. RDP classifier software was used for species comparison annotations, and annotation results with a confidence interval greater than 0.7 were retained. Blast software was used for species comparison annotations.

### Statistical Methods

SPSS (Version 19.0) was used for statistical analysis and graph plotting. Chi-square test and Fisher’s test were adopted for the categorized data. *t*-test or non-parametric test was used for continuous data and the test level was α = 0.05.

### Ethical Approval and Patient Informed Consent

This study was approved by the ethics committee of the hospital. The sampling process and detail research plan were understood and an informed consent form was signed by all subjects.

## Results

### General Information

In this study, 22 cases of gastric cancer patients were included in the gastric cancer group. The diagnosis under gastroscopy and histopathology was clear, without surgery, radiotherapy, and chemotherapy. Among them, the pathological diagnosis includes 11 cases of gastric antrum adenocarcinoma, 4 cases of gastric cardia adenocarcinoma, and 4 cases of gastric cancer in the corner of the stomach. There were 2 cases of gastric antrum carcinoma in situ and 1 case of gastric cancer in the corner of the stomach in situ. Meanwhile, 30 cases were included in the healthy group, with no digestive system symptoms, negative clinical tests, and normal gastric mucosa by gastroscopy. The age of the gastric cancer group was 60.68 ± 9.574 (38–78 years old), while the age of the healthy group was 58.87 ± 15.520 (14–92 years old). There was no statistical difference between the groups, *P* = 0.630. The ratio of men to women in the gastric cancer group was 2.7:1, and in the healthy group was 1:1. There was no statistical difference between the groups, *P* = 0.099. The gastric cancer group and the healthy group are mainly composed of Han ethnicity. See Table 1.

**Table 1:** Comparison of general information between gastric cancer and healthy groups

Group	Number	Age (years)	Gender (Male/Female)
Gastric cancer	22	60.68 ± 9.574	16/6
Healthy	30	58.87 ± 15.520	15/15
t/χ^2^		0.484	2.723
*p*		0.630	0.099

### Diversity Analysis of Intestinal Flora in Gastric Cancer Group and Healthy Group

**Fig. 2:**
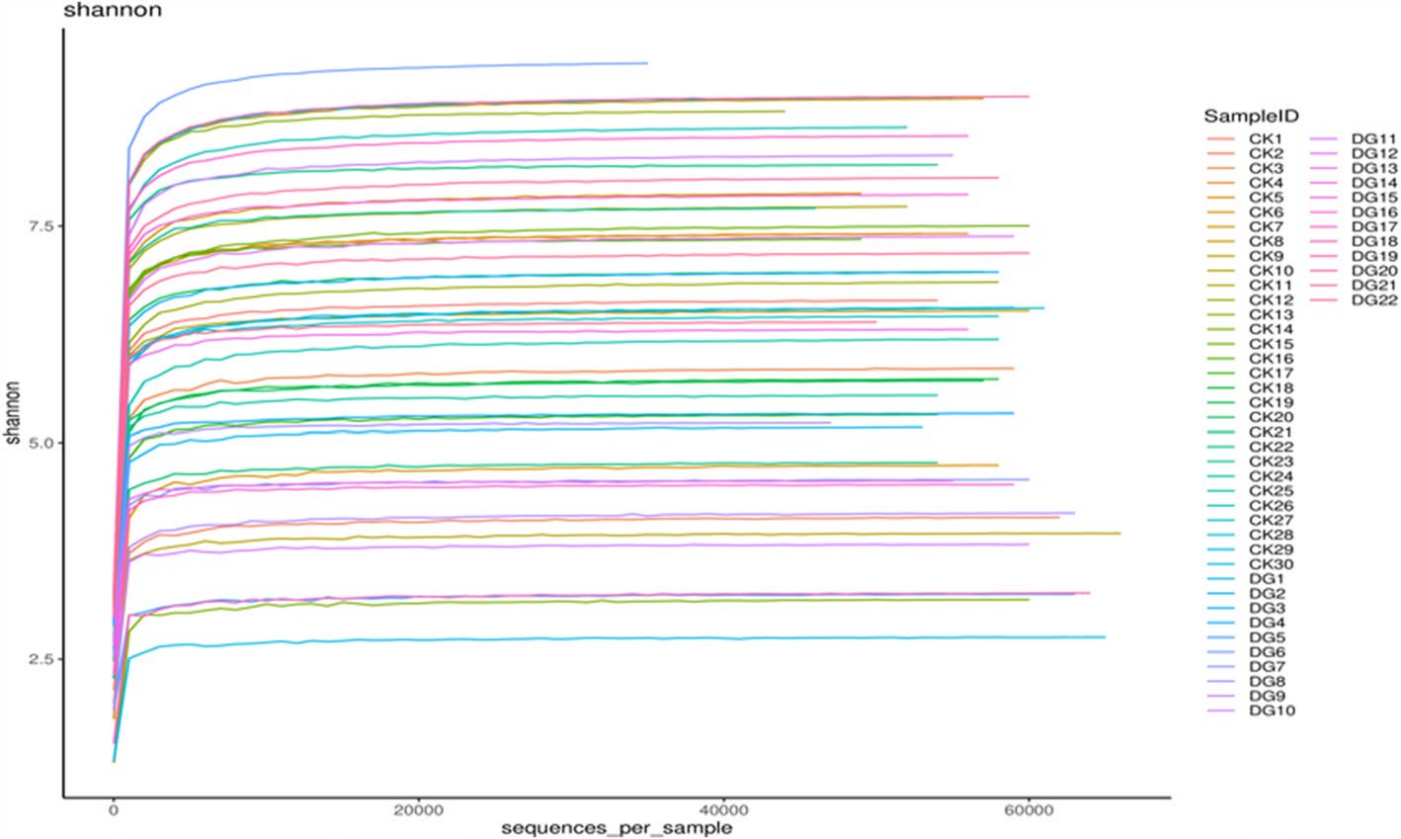
Dilution curve is used to assess the abundance and sequencing depth of the fecal and intestinal flora of the gastric cancer group and the healthy group. The y-axis represents the number of OTUs, and the x-axis represents the number of randomly selected sequencing sequences (DG: gastric cancer group; CK: healthy group)

**Fig. 3:**
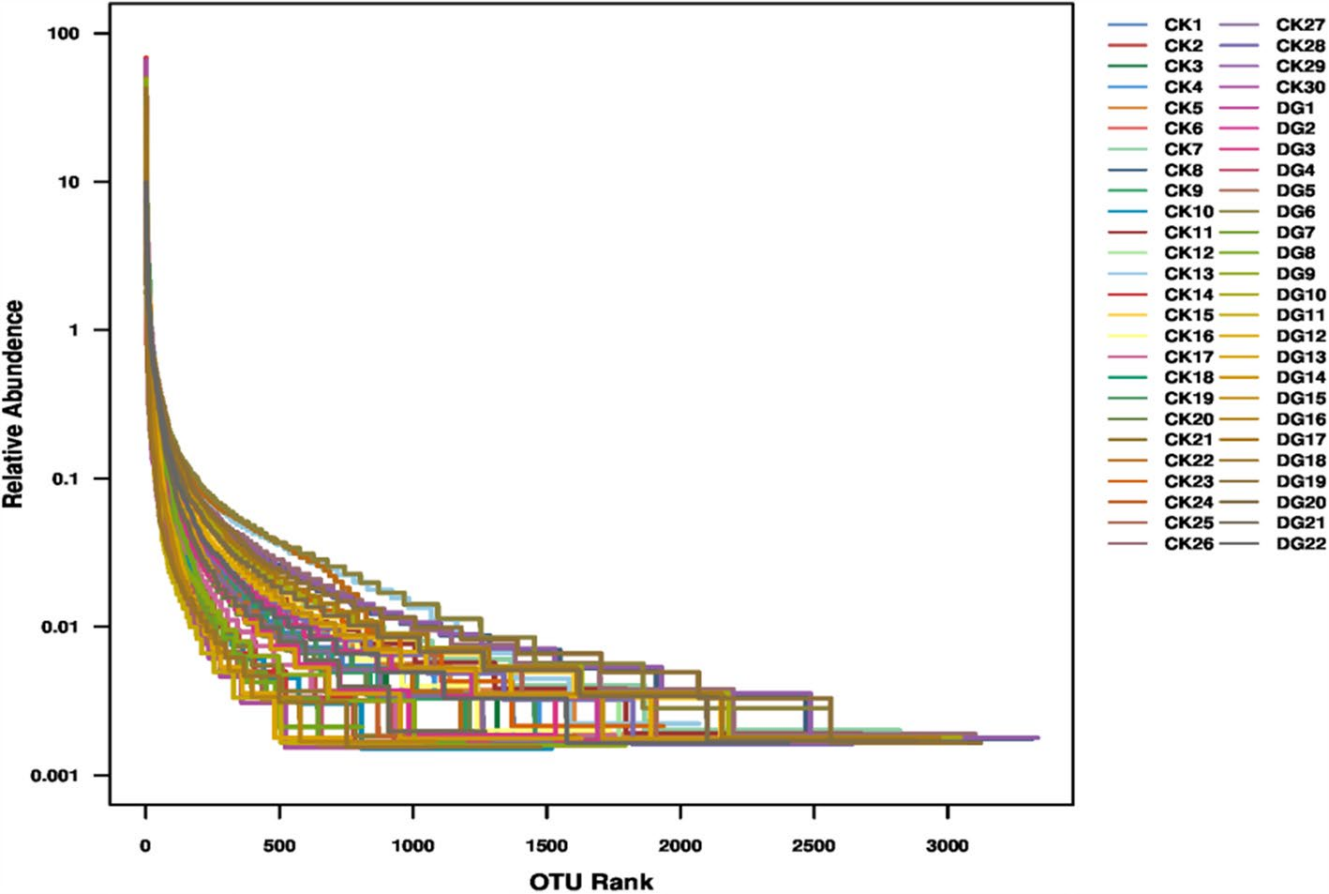
Rank-abundance curve diagram of gastric cancer group and healthy group. The y-axis represents the abundance of OTUs, and the x-axis represents the sequence number of the corresponding abundance of OTUs (DG: gastric cancer group; CK: healthy group)

**Fig. 4:**
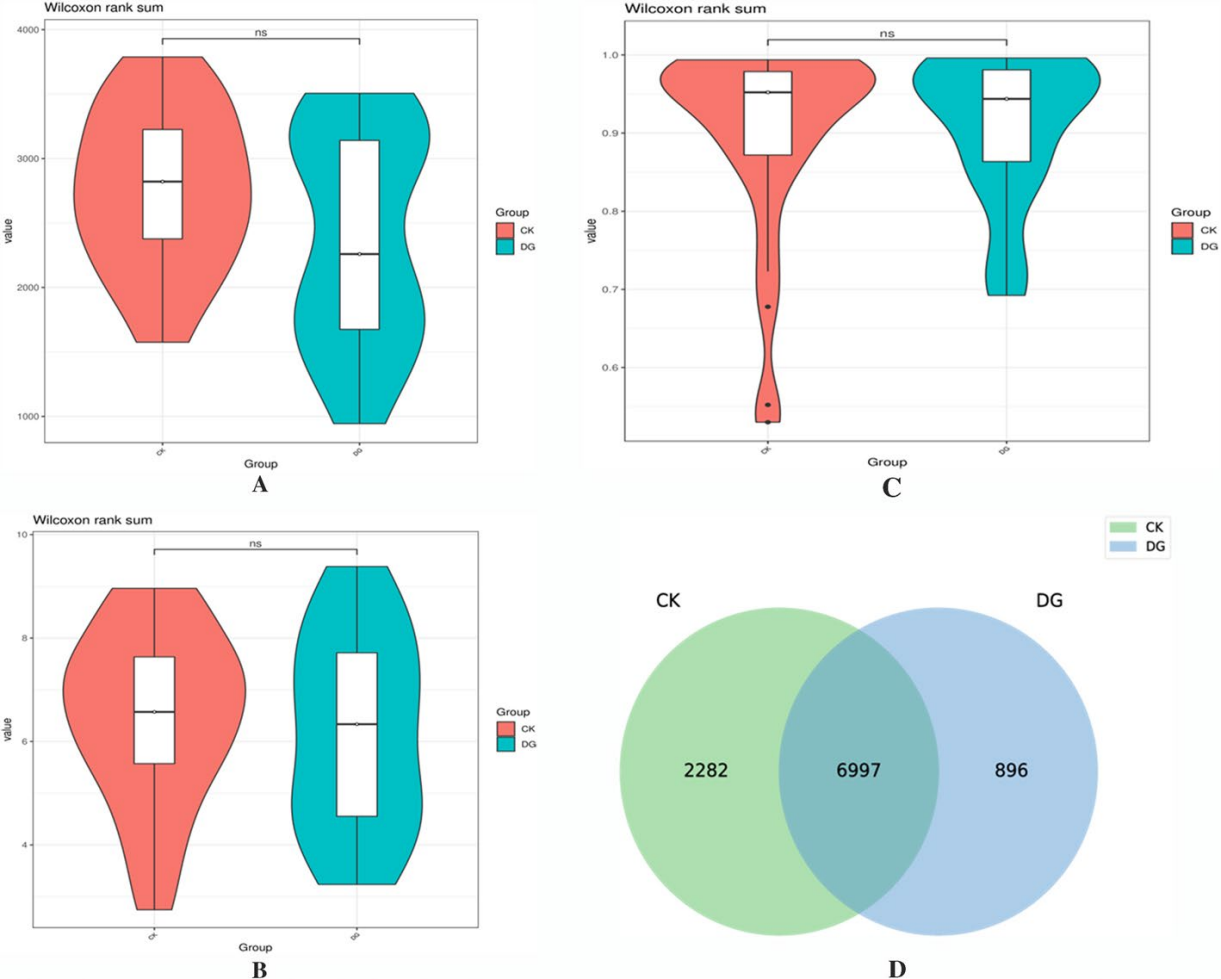
Comparative analysis of the abundance and diversity of fecal and intestinal flora between the gastric cancer group and the healthy group (CK, healthy group; DG, gastric cancer group). **A** (Chao1), **B** (Shannon), **C** (Simpson), **D** (Venn)

In order to avoid the diversity differences between different samples that may lead to errors in species diversity, this study has evaluated the diversity of intestinal flora and the depth of sequencing through the analysis of the dilution curve (Fig. 2). A total of 52 samples were used for 16S rDNA gene sequencing. After applying the split_libraries software to perform quality control in QIIME, the data volume of clean tags is distributed between 55,229 and 72,806, and the clean tags are removed by UCHIME software to remove the chimera, and the data volume of valid tags (that is, the final data used for analysis) is distributed between 35,313 and 66,112. The average length of valid tags is ranging from 376.82 to 421.39 bp, and the number of OTUs in each sample is ranging from 766 to 3335. Shannon exponential dilution curve suggests that the sequencing depth is reasonable. The rank-abundance curve is used to evaluate richness and evenness of the bacterial species in different samples (Fig. 3). The figure shows that the horizontal direction of each sample curve forms a “long tail,” indicating that most of the OTUs in each sample are at a low abundance level, and the evenness of the flora composition is reasonable. The Chao1, Shannon, and Simpson indexes are calculated based on the OTU species and abundance. The coverage of each sample in the gastric cancer group and the healthy group is greater than 98%, indicating that the microbial species detected in this sequencing can cover most of the species in the sample. It is consistent with the results of Shannon’s exponential dilution curve. The Chao1 index reflects the abundance of species in the sample, that is, the number of OTUs. A larger Chao1 value represents a larger total number of species, indicating a higher richness (see Fig. 4A). The Chao1 index of the gastric cancer group was 2381.28 ± 823.68, and that of the healthy group was 2750.23 ± 618.10. Compared with the healthy group, the species richness of the gastric cancer group was *t* = 1.768 (*P* = 0.085), and the difference was not statistically significant. The Shannon and Simpson indices were used to reflect the diversity of the community, including species abundance and species evenness. The larger the Shannon and the smaller the Simpson indices, the higher the species diversity in the sample. The results showed that the median Shannon index of the gastric cancer group was 6.156 ± 1.89, and that of the healthy group was 6.463 ± 1.67. Compared with the healthy group, the species diversity of the gastric cancer group was *t* = 0.619 (*P* = 0.539), and the median Simpson index of the gastric cancer group was 0.908 ± 0.09. The healthy group was 0.896 ± 0.13, and the difference was not statistically significant *t* = −0.352 (*P* = 0.726) (see Fig. 4C). In the figure, the number of common OTU units in the gastric cancer group and the healthy group was 6997, the number of unique OTU units in the healthy group was 2282, and the number of unique OTU units in the gastric cancer group was 896, the difference was statistically significant (*χ*^2^ = 495.829, *P* < 0.000) (see Fig. 4D).

### Composition and Abundance Distribution of Intestinal Flora in Gastric Cancer Group and Healthy Group

**Fig. 5:**
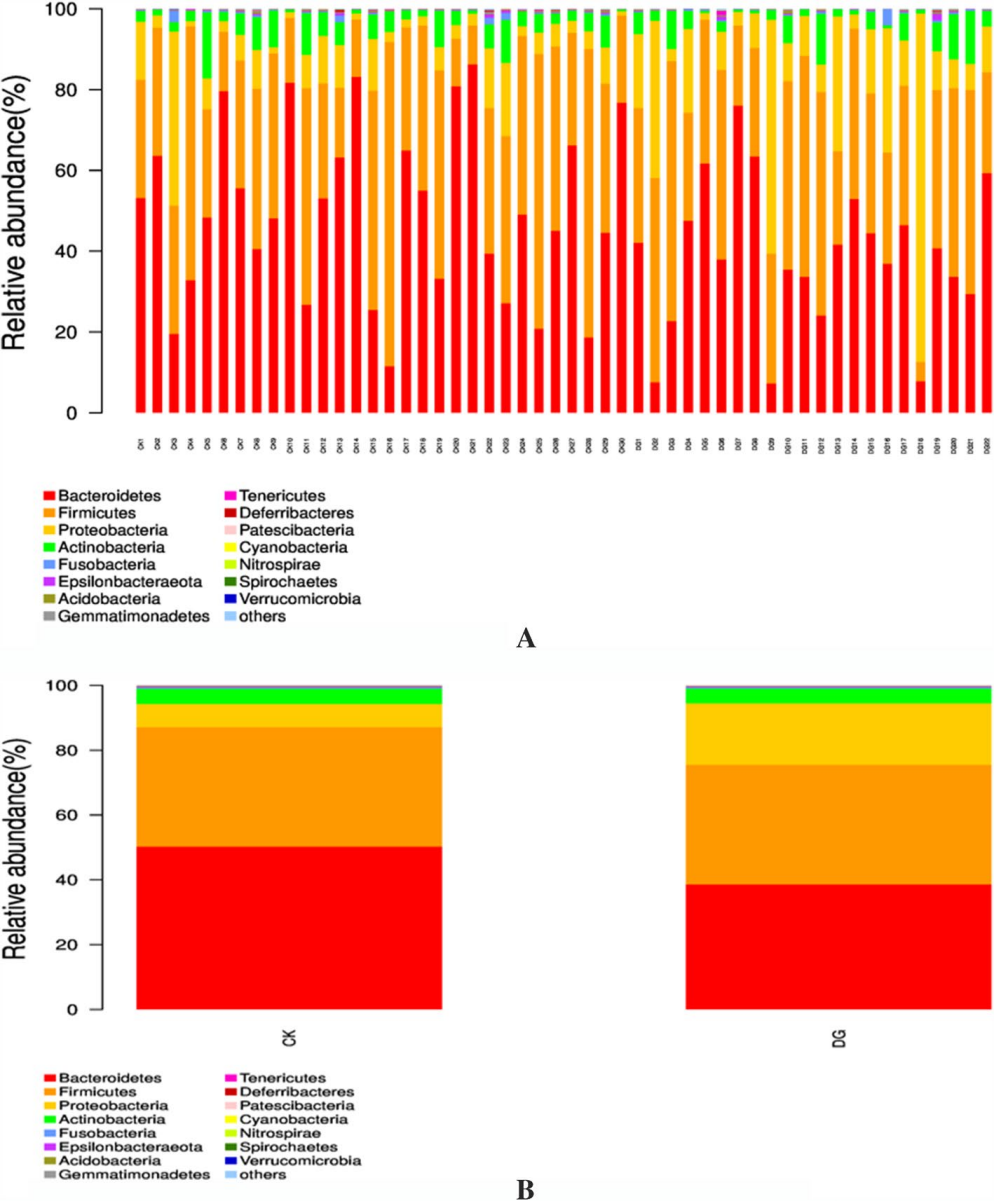
The compositional level of fecal intestinal flora in the gastric cancer group and the healthy group (top 15 dominant bacteria at the phylum level)

At the phylum and genus levels, the abundance of the gastric cancer group and the healthy group samples were evaluated. At the phylum level, the top 15 dominant bacteria in the healthy group are *Bacteroidetes* (49.78%), Firmicutes (37.03%), Proteobacteria (7.27%), Actinobacteria (4.82%), Fusobacteria (0.42%), Epsilonbacteraeota (0.17%), Acidobacteria (0.16%), Gemmatimonadetes (0.09%), Tenericutes (0.06%), Deferribacteres (0.05%), Patescibacteria (0.02%), Cyanobacteria (0.01%), Nitrospirae (0.01%), Spirochaetes (0.01%), and Verrucomicrobia (0.01%). The top 15 dominant bacteria in the gastric cancer group are as follows: Bacteroidetes (38.74%), Firmicutes (37.34%), Proteobacteria (18.34%), Actinobacteria (4.56%), Fusobacteria (0.40%), Epsilonbacteraeota (0.20%), Acidobacteria (0.13%), Tenericutes (0.07%), Gemmatimonadetes (0.07%), Deferribacteres (0.03%), Patescibacteria (0.02%), Cyanobacteria (0.01%), Nitrospirae (0.01%), Spirochaetes (0.01%), and Verrucomicrobia (0.01%). Bacteroidetes and Firmicutes had the highest abundance in the two groups, in which the percentage of Bacteroides in the healthy group was 49.78%, and the percentage in the gastric cancer group was 38.74%, and the difference was not statistically significant (*P* = 0.224). Meanwhile, the percentage of Firmicutes in the healthy group was 37.03% and the percentage in the gastric cancer group was 37.34%, and the difference was not statistically significant (*P* = 0.288). The percentage of Proteobacteria in the healthy group was 7.27% and the percentage in the gastric cancer group was 18.34%, and the difference was statistically significant (*P* = 0.003). Actinobacteria in the healthy group and gastric cancer group accounted for 4.82% and 4.56%, respectively, and the difference was not statistically significant (*P* = 0.512). The Fusobacteria in the healthy group and gastric cancer group was 0.42% and 0.40%, respectively, and the difference was not statistically significant (*P* = 0.488). Epsilonbacteraeota in the healthy group and gastric cancer group was 0.17% and 0.20%, respectively, and the difference was not statistically significant (*P* = 0.849). Acidobacteria in the healthy group and gastric cancer group was 0.16% and 0.13%, respectively, and the difference was not statistically significant (*P* = 0.281); see Table 2 and Fig. 5.

**Table 2:** Comparison of intestinal flora levels between gastric cancer group and healthy group (top 15 flora)

Group	Gastric cancer	Healthy	*t/Z*	*p*
Intestinal flora				
Bacteroidetes	0.38 ± 0.181	0.50 ± 0.217	1.942	0.224
Firmicutes	0.37 ± 0.140	0.37 ± 0.182	− 0.065	0.288
Proteobacteria	0.183 ± 0.205	0.073 ± 0.082	− 2.681	0.003
Actinobacteria	0.046 ± 0.040	0.05 ± 0.037	0.235	0.512
Fusobacteria	0.002 (0.001, 0.003)	0.002 (0.001, 0.004)	− 0.463	0.643
Epsilonbacteraeota	0.0008 (0.0003, 0.0019)	0.0011 (0.0004, 0.0016)	− 0.852	0.349
Acidobacteria	0.0013 ± 0.0013	0.0016 ± 0.0010	1.021	0.488
Gemmatimonadetes	0.0007 ± 0.0007	0.0009 ± 0.0009	0.971	0.849
Tenericutes	0.0007 ± 0.0021	0.0006 ± 0.0007	− 0.304	0.281
Deferribacteres	0.0001 (0.0001, 0.003)	0.0001 (0.00006, 0.0005)	− 0.797	0.425
Patescibacteria	0.0002 ± 0.0002	0.0002 ± 0.0002	0.237	0.425
Cyanobacteria	0.0001 ± 0.0001	0.0001 ± 0.0002	0.215	0.907
Nitrospirae	0.0001 ± 0.0001	0.0001 ± 0.0001	0.933	0.630
Spirochaetes	0.0001 ± 0.0001	0.0001 ± 0.0001	0.479	0.790
Verrucomicrobia	0.0000 (0.0000, 0.0001)	0.0000 (0.0000, 0.0001)	− 0.067	0.947

**Fig. 6:**
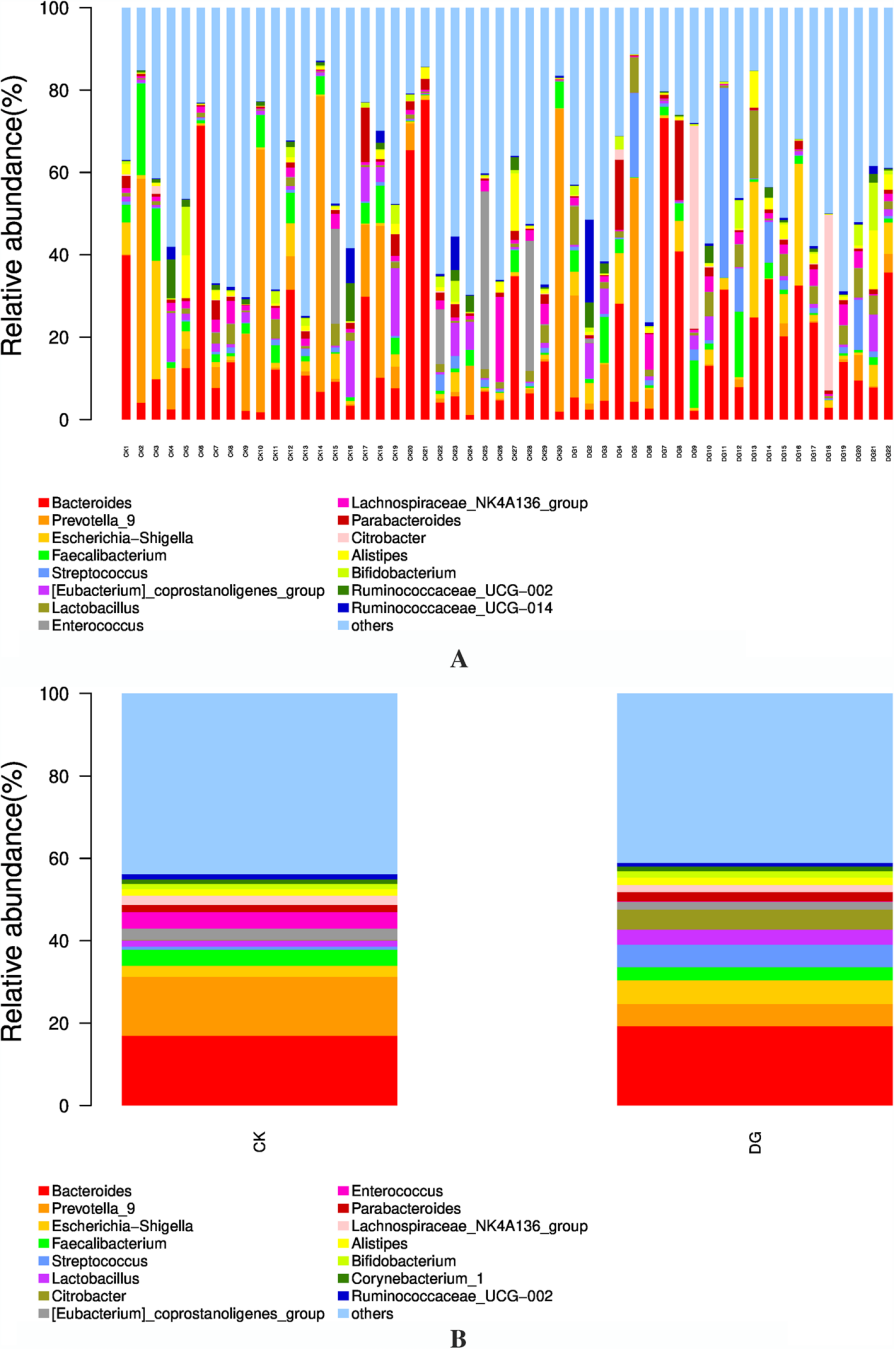
The compositional level of fecal intestinal flora in the gastric cancer group and the healthy group (top 15 dominant bacteria at the genus level)

At the genus level, the top 15 dominant bacteria in the healthy group are mainly *Bacteroides* (16.98%), *Prevotella_9* (13.21%), *Escherichia-Shigella* (2.66%), *Faecalibacterium* (3.74%), *[Eubacterium]_coprostanoligenes_group* (2.86%), *Enterococcus* (3.79%), *Lachnospiraceae_NK4A136_group* (2.32%), *Parabacteroides* (1.81%), and *Alistipes* (1.63%). The top 15 dominant bacteria in the gastric cancer group are mainly *Bacteroides* (19.14%), *Escherichia-Shigella* (5.76%), *Streptococcus* (5.22%), *Prevotella_9* (5.10%), *Citrobacter* (4.35%), *Lactobacillus* (3.64%), *Faecalibacterium* (3.10%), *Parabacteroides* (2.40%), *[Eubacterium]_coprostanoligenes_group* (1.92%), *Alistipes* (1.89%), *Lachnospiraceae_NK4A136_group* (1.83%), and *Bifidobacterium* (1.59%). There is a difference in the composition of intestinal flora between the gastric cancer group and the healthy group. The main dominant floras of the healthy group are *Bacteroides* (16.98%) and *Prevotella_9* (13.21%), while the main dominant flora of the gastric cancer group is only the *Bacteroides* (19.14%). Compared with the healthy group, the gastric cancer group had statistically significant differences in *Prevotella_9*, *Streptococcus*, and *Lactobacillus* (*P* = 0.014, *P* < 0.001, *P* < 0.001). See Table 3 and Fig. 6.

**Table 3:** Comparison of the intestinal flora levels between the gastric cancer group and the healthy group (top 15 flora)

Group	Gastric cancer	Healthy	*t/Z*	*p*
Intestinal flora				
*Bacteroides*	0.135 (0.045, 0.318)	0.084 (0.041, 0.181)	− 0.945	0.345
*Prevotella_9*	0.05 ± 0.123	0.13 ± 0.226	1.525	0.014
*Escherichia-Shigella*	0.226 (0.006, 0.072)	0.009 (0.004, 0.026)	− 1.574	0.115
*Faecalibacterium*	0.03 ± 0.043	0.04 ± 0.047	0.500	0.838
*Streptococcus*	0.052 ± 0.105	0.008 ± 0.009	− 2.306	0.000
*[Eubacterium]_coprostanoligenes_group*	0.019 ± 0.028	0.029 ± 0.441	0.878	0.117
*Lactobacillus*	0.036 ± 0.041	0.015 ± 0.015	− 2.639	0.000
*Enterococcus*	0.0009 (0.0003, 0.0015)	0.0008 (0.0005, 0.0022)	− 1.019	0.308
*Lachnospiraceae_NK4A136_group*	0.018 ± 0.021	0.023 ± 0.037	0.553	0.787
*Parabacteroides*	0.024 ± 0.515	0.018 ± 0.025	− 0.540	0.123
*Citrobacter*	0.0002 (0.0001, 0.0010)	0.0005 (0.0002, 0.0016)	− 1.556	0.120
*Alistipes*	0.016 ± 0.030	0.019 ± 0.034	0.519	0.778
*Bifidobacterium*	0.013 ± 0.024	0.016 ± 0.028	0.444	0.719
*Ruminococcaceae_UCG-002*	0.013 ± 0.024	0.009 ± 0.016	0.443	0.487
*Ruminococcaceae_UCG-014*	0.0037 (0.0018, 0.0052)	0.0025 (0.0003, 0.0059)	− 0.741	0.459

**Fig. 7:**
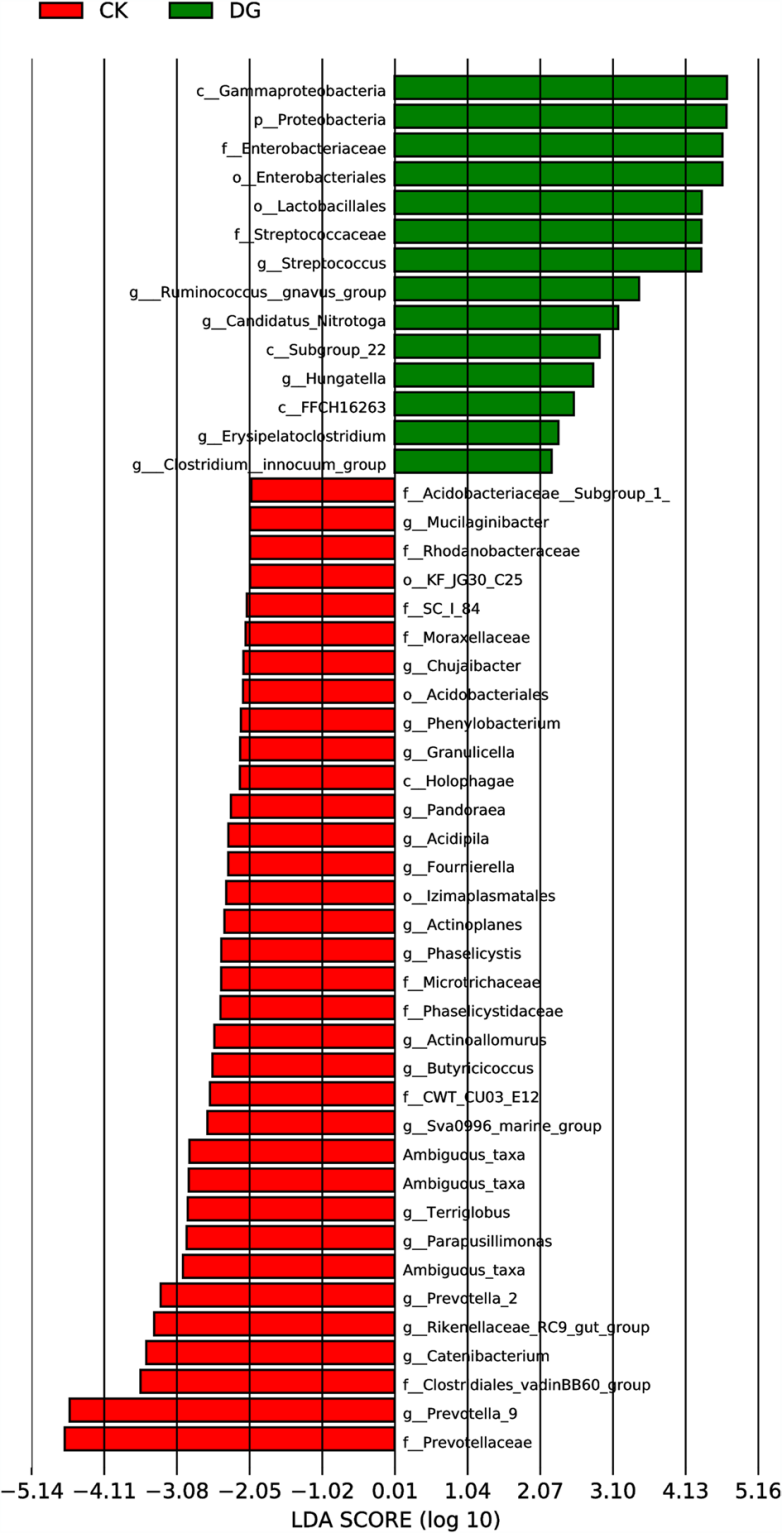
LEfSe analysis diagram of the structural difference in fecal and intestinal flora between gastric cancer group and healthy group (CK, healthy group; DG, gastric cancer group. The red line indicates the relatively high abundance species in the healthy group, and the green line indicates the relatively high abundance species in the gastric cancer group. The letters in front of the species indicate different levels. p represents the phylum level, c represents the class level, o represents the order level, f represents the family level, g represents the genus level, and s represents the species level)

In order to further analyze the composition of the intestinal flora and the difference in the abundance of its component at each taxonomic levels of phyla, class, order, family, and genus, and at the same time to screen the dominant flora of the intestinal microbial communities in the gastric cancer and healthy control groups, we have adopted the metagenomics analysis method to screen out the characteristic of dominant intestinal flora in the two groups. The LEfSe analysis of the structural differences of the fecal and intestinal flora between the gastric cancer and healthy groups shows that the most dominant flora in the healthy group is *Prevotella_9*, *Prevotellaceae*, and *Clostridiales_vadinBB60_group*, whereas the dominant flora in the gastric cancer group is Gammaproteobacteria, Enterobacteriales, Proteobacteria, and *Streptococcus*. See Fig. 7

## Discussion

In recent years, increasing number of researchers have conducted in-depth research on the intestinal flora and found that different regions have a relatively vital effect on the changes in the intestinal flora [3, 4]. There are studies on more than 7000 in different geographic regions of Guangdong Province. It was found that the composition and diversity of intestinal flora in different geographical populations are different [5, 6]. Gastric cancer is one of the malignant tumors with the highest incidence in the Qinghai Plateau. Qinghai Province is located in the Qinghai-Tibet Plateau with a typical high-altitude and low-oxygen environment. Studies have found that various factors such as gastrointestinal oxygen concentration, intestinal pH, and drug may change the diversity of intestinal flora [5], suggesting that hypoxic environment may have an impact on the diversity and composition of the intestinal flora. Residents who have lived in this area for a long time tend to smoke and drink, and consuming with foods high in salt and fat, more meat, and less vegetable. At present, most studies on gastric cancer in plateau areas focus on factors such as immunity and genetics, while there is relatively less study on the intestinal flora of patients with gastric cancer. Therefore, in this study, the compositional characteristics of the intestinal flora in healthy and gastric cancer patients in the plateau area were analyzed. By exploring the structural difference and diversity of the intestinal flora in both gastric cancer patients and healthy patients in the plateau area, we aim to determine the pathogenic factors of gastric cancer in plateau areas from the perspective of intestinal flora, hence further provide new ideas for the prevention and precise treatment of gastric cancer in plateau areas.

The results of the analysis of the abundance and evenness of the intestinal flora show that in the analysis of the intestinal flora diversity, the composition and diversity of the intestinal flora of the gastric cancer group are different from those of the healthy group, but it was not statistically significant. It is inconsistent with the significant difference in the intestinal flora diversity between gastric cancer patients and healthy people reported in relevant domestic studies [7, 8]. This can be probably due to the geographical factors, environment, and eating habits of the population in the plateau area. In the Venn diagram, the number of OTU units shared by the gastric cancer group and the healthy group is 6997, the number of unique OTU units in the healthy group is 2282, and the number of unique OTU units in the gastric cancer group is 896, which is also statistically significant, suggesting a significant reduced of flora diversity in gastric cancer in plateau areas, which is consistent with the results of related studies [9].

At the phylum level, the results of the intestinal flora composition and abundance distribution show that Bacteroidetes and Firmicutes have the highest abundance in both the healthy and gastric cancer groups, followed by the *Proteobacteria*, *Actinobacteria*, *Fusobacteria*, *Epsilonbacteraeota*, and *Acidobacteria*. As for *Proteobacteria*, its abundance in the gastric cancer group is significantly higher than the healthy group, the difference is statistically significant. This result is consistent with the analysis of the relevant flora in gastric cancer patients reported by Liang Weiren [10] and Qi Yufeng [11]. This suggests that Proteobacteria can be an important parameter in multi-omics analysis on the next step or a pathogenic factor that will be further explored and studied.

At the genus level, the composition of the intestinal flora between the gastric cancer group and the healthy group is different. *Bacteroides* and *Prevotella_9* are the main dominant bacteria in the healthy group, while the only main dominant bacteria in the gastric cancer group is *Bacteroides*. Compared with the healthy group, *Prevotella_9* in the gastric cancer group was significantly reduced, while the *Streptococcus* and *Lactobacillus* were significantly increased. It is consistent with the results reported by E Dias-Jácome [12] and Ferreira RM [13]. However, related studies by Nakamoto et al. [14] found that *Lactobacillus* can exert potential anti-tumor effects by enhancing its immune tolerance, suggesting that *Lactobacillus* could be an important parameter and is warranted for further study in the intestinal flora of gastric cancer patients. Since this characteristically significant intestinal flora are highly abundant in the feces of gastric cancer patients, it is necessary to further investigate the enriched bacteria in the intestinal flora of patients found in this study to identify the intestinal flora with non-invasive potential in diagnosing gastric cancer. It is worth mentioned that the proportion of *Helicobacter pylori* in stool samples of gastric cancer patients is very low and is consistent with previous literature review. This indicates that human intestinal environment is not suitable for the survival of *Helicobacter pylori* [15, 16].

Although this study has examined the intestinal flora diversity of patients with gastric cancer and healthy people in plateau areas, and has suggested different characteristics between gastric cancer patients and intestinal flora in relation with geographical region, there are also limitations. Firstly, the composition, diversity, and complicated functions of the intestinal flora, thus the results of the study might be affected by the environment, diet, and metabolism which then may cause bias in data collection. Secondly, statistical insignificant result might be due to the small sample size of this study will needs to be further evaluated using a much larger sample size. Lastly, despite the diversity and abundance of the flora were determined, the functional study has yet to complete.

Relevant studies have shown that maintaining the balance of intestinal flora in patients with gastrointestinal malignant tumors through intervention can inhibit the occurrence of tumors and improve their prognosis [17]. Therefore, a comprehensive study of the relationship between the intestinal flora and the occurrence and development of gastric cancer in plateau areas is of great significance in the early diagnosis, prevention, and treatment of patients. This study initially investigated the diversity and composition of the intestinal flora of gastric cancer patients and healthy people under the hypoxic environment of Qinghai Plateau has provided a foundation for further research on the intestinal flora of gastric cancer patients in the plateau area. However, the sample size of the existing research is relatively small, and the research has not been associated with the related factors including eating habits and ethnic composition. In the next step, we plan to expand the sample size and include all samples into the metagenomics and metabolomics studies by considering the association with phenotypic characteristics, eating habits, and family history in order to better delineate the expression profile of the intestinal flora of gastric cancer patients in the plateau area. This will explore the diagnostic markers of the intestinal flora of gastric cancer patients in the plateau area, and finally reduce the incidence of gastric cancer in the plateau area through early flora intervention, thus providing new ideas for the diagnosis and treatment of gastric cancer patients.

## Data Availability

Not applicable.
