# Correction to: Study on the Characteristics of Intestinal Flora Composition in Gastric Cancer Patients and Healthy People in the Qinghai-Tibet Plateau

**DOI:** 10.1007/s12010-022-03874-z

**Published:** 2022-03-25

**Authors:** Zilong Zhang, Linghong Zhu, Yanqing Ma, Bo Wang, Caihong Ci, Jingni Zhang, Yunsong Zhou, Chunjiang Dou, Qiaoling Gu, Yan An, Yongmei Lan, Jin Zhao

**Affiliations:** 1 Northwest Minzu University, Lanzhou, 730030 Gansu Province China; 2 Department of Oncosurgery, Qinghai Provincial People’s Hospital, Xining, 810007 Qinghai Province China; 3 Department of Science and Education, Qinghai Provincial People’s Hospital, Xining, 810007 Qinghai Province China; 4 Key Laboratory of Environmental Ecology and Population Health in Northwest Minority Areas, Northwest Minzu University, Lanzhou, 730030 Gansu Province China

**Correction to: Applied Biochemistry and Biotechnology**

10.1007/s12010-021-03732-4

The original article has been corrected. The correct author position and new acknowledgements should be:

- Yongmei Lan and Jin Zhao contributed equally.They are corresponding authors.
- Zilong Zhang^1,2^, Linghong Zhu^3^, Yanqing Ma^1^, Bo Wang^3^, Caihong Ci^1^, Jingni Zhang^3^, Yunsong Zhou^1^,Chunjiang Dou^1^, Qiaoling Gu^1^,Yan An^1^, Yongmei Lan^1*^, Jin Zhao^1,4*^

**Acknowledgements**

1. Study on the relationship between ethnic characteristics, environmental factors, and disease spectrum of Gansu endemic minorities, the project of cultivating innovative team of Northwest University for Nationalities funded by the basic scientific research business of central universities (project number 31920190030), and the key project of Northwest University for Nationalities funded by the basic scientific research business of central universities.
2. Health and Family Planning Guidance Project of Qinghai Health and Family Planning Commission 2019 (Project No. 2019-wjzdx-41).
3. Cell culture innovation team. Project source: school-level innovation team construction project in 2020 (project approval number: 10013649).

